# Robots, ledgers, and RevPAR: a blockchain-enabled AI–robotics conceptual model for sustainable hotel revenue and asset management

**DOI:** 10.3389/frobt.2026.1779342

**Published:** 2026-03-03

**Authors:** Leonard A. Jackson

**Affiliations:** Leven School of Management Entrepreneurship and Hospitality, Kennesaw State University, Kennesaw, GA, United States

**Keywords:** artificial intelligence, blockchain, dynamic pricing, hotel asset management, real estate, revenue management, service robots, sustainability

## Abstract

**Introduction:**

Robotics and artificial intelligence (AI) are rapidly reshaping hospitality by automating frontline and back-of-house processes, augmenting service encounters, and expanding the analytical scope of revenue management. Yet, existing research remains fragmented: service-robot studies largely emphasize adoption and human–robot interaction, while revenue-management research prioritizes pricing and distribution, sustainability research focuses on environmental practices, and hotel real-estate scholarship foregrounds governance and asset value. Meanwhile, blockchain technologies—through distributed ledgers, smart contracts, digital identity, and tokenization—offer a complementary trust and value-transfer layer that can address coordination and verification problems across hotel ecosystems (e.g., data sharing, sustainability claims, and owner–operator contracting).

**Methods:**

Drawing on an integrative literature synthesis, this conceptual article develops an integrative framework linking AI–robotics and blockchain capabilities to three interdependent hotel decision domains: (1) revenue management (demand forecasting, dynamic/open pricing, channel and loyalty optimization), (2) sustainability and operations (resource optimization, waste circularity, predictive maintenance), and (3) real estate and hotel asset management (digital twins, CapEx planning, valuation and risk analytics, and tokenized financing).

**Results:**

A conceptual model is proposed in which AI–robotics and blockchain jointly build digital operational and market-intelligence capabilities that improve financial performance (RevPAR/GOPPAR and net operating income), sustainability performance (carbon and resource intensity), and long-term asset value. Ten propositions articulate mechanisms and boundary conditions related to governance, ethics, privacy, cybersecurity, organizational readiness, regulation, and market context.

**Discussion:**

The article concludes with implications for hotel managers, owners, investors, and researchers, and outlines a future research agenda for hospitality, tourism, service management, and real-estate scholars.

## 1 Introduction

Hospitality has traditionally been characterized as a labor-intensive industry in which value is co-created through service encounters, experiential design, and intensive coordination among guests, employees, brands, owners, and technology intermediaries. Over the last decade, however, hotels have entered a phase of accelerated automation and datafication. Service robots, AI-driven analytics, and smart infrastructures are increasingly deployed not only to reduce costs but also to reconfigure the service experience and the information foundations of managerial decision making (Belanche et al., 2020; Ivanov et al., 2019; Wirtz et al., 2018).

Research in hospitality and tourism has documented a proliferation of robotic applications ranging from delivery and cleaning robots to socially interactive concierge agents. Literature reviews highlight that scholarly attention has concentrated on adoption drivers, attitudes, and human–robot interaction (HRI), reflecting the novelty of robots in guest-facing contexts (Ivanov et al., 2019). Empirical studies emphasize that trust, perceived safety, and the quality of the interaction strongly shape customer evaluations, suggesting that robotics adoption is as much a service-design challenge as a technology choice (Park, 2020; Tung and Au, 2018; Tussyadiah et al., 2020).

At the same time, hotel decision making has become more data intensive and increasingly algorithmic. “Smart hospitality” scholarship argues that interconnectivity and interoperability among property management systems, distribution platforms, IoT sensors, and analytics enable value creation in a multi-actor ecosystem (Buhalis and Leung, 2018; Gretzel et al., 2015; Buhalis et al., 2023). Big-data research demonstrates that text analytics applied to online reviews can reveal experiential attributes linked to satisfaction and provide actionable insights for operational and marketing decisions (Xiang et al., 2015). Systematic reviews further show that hospitality analytics has expanded rapidly, but that the field still needs stronger theory integration and clearer links to strategic outcomes (Mariani and Baggio, 2022).

These technological shifts matter because the hotel business model is uniquely sensitive to information and coordination. Hotel rooms are perishable, capacity is fixed in the short run, and demand fluctuates across seasons, days of week, and market segments. Revenue management therefore became central to hotel strategy, with classic yield-management principles focusing on allocating capacity to the right customer at the right price (Kimes, 1989). Over time, revenue management evolved beyond room inventory to encompass multiple revenue streams and strategic alignment with marketing and operations (Cross et al., 2009). More recent work argues that a move toward open pricing and individualized offers is facilitated by the growing availability of demand signals and customer-level data (Talón-Ballestero et al., 2022).

However, revenue optimization is increasingly constrained by behavioral and ethical considerations. Dynamic pricing can trigger perceptions of unfairness, particularly when customers see large price differences across comparable stays (Alderighi et al., 2022). Online contexts also shape customer evaluations; price presentation strategies influence willingness to book, suggesting that the psychology of pricing is inseparable from algorithmic optimization (Noone and Mattila, 2009). These findings imply that the future of revenue management will depend not only on better algorithms but also on transparency, governance, and trust.

Sustainability adds another strategic dimension. Hotels have implemented a wide range of environmental initiatives, but adoption and sophistication vary across markets. Survey research in Europe indicates that operators recognize the importance of environmental stewardship yet face economic and institutional constraints (Bohdanowicz, 2006). In multinational chains, environmental programs have been linked to corporate governance and HR practices, demonstrating that sustainability is embedded in organizational systems rather than isolated practices (Bohdanowicz et al., 2011). From the demand side, sustainable practices can influence guest satisfaction and intention to return (Berezan et al., 2013), while systematic reviews call for research that connects environmental strategies to innovation and competitive positioning (Elkhwesky, 2022).

Crucially, hotels are not only operating businesses but also real estate assets. Owners and investors evaluate properties based on expected net operating income (NOI) and risk, and governance arrangements often separate real estate ownership from hotel operations. The profession of hotel asset management emerged to align operator decisions with owner value objectives and has evolved into a continuous value-maximization role (Singh et al., 2012). Asset management perspectives also highlight that performance metrics matter: analyses comparing RevPAR and GOPPAR show that revenue and profit indicators can diverge at both property and firm levels, affecting how operational decisions translate into value (Lee et al., 2019).

Blockchain technologies introduce a further layer of potential disruption. Tourism scholarship suggests that blockchain can support new forms of disintermediation, transparency, and governance by enabling immutable records and programmable rules (Önder and Treiblmaier, 2018). Commentaries frame blockchain as a potential watershed for tourism development, with implications for payments, loyalty, identity, and coordination (Kwok and Koh, 2019). Yet, hospitality research has rarely connected blockchain’s trust infrastructure to the AI-enabled automation and analytics that hotels are adopting. This is a missed opportunity because AI increases data volume and decision autonomy, while blockchain can provide auditability, consent management, and contract automation—capabilities that become increasingly valuable under algorithmic operations.

This article addresses this gap by developing a conceptual model that integrates AI–robotics and blockchain in hospitality with specific focus on revenue management, sustainability, and real estate/hotel asset management. It is proposed that these technologies jointly build digital operational and market-intelligence capabilities that improve financial performance, sustainability performance, and long-term asset value, while being subject to boundary conditions related to governance, ethics, privacy, cybersecurity, organizational readiness, regulation, and market context. The model is intended to support theory development and to guide future empirical research and managerial practice in the global hospitality context. Specifically, the model’s primary theoretical contribution is to foreground digital operational and market-intelligence capability as the causal mechanism that converts AI–robotics and blockchain resources into sustainable value creation (financial performance, environmental performance, and long-term asset value) in complex, multi-actor hotel service ecosystems, extending prior smart hospitality and digital transformation frameworks.

## 2 Literature review

### 2.1 Service robots and AI in hospitality service ecosystems

Service robots can be defined as system-based autonomous and adaptable interfaces that interact, communicate, and deliver service to an organization’s customers or employees. In hospitality, robots increasingly operate in public spaces and interact directly with guests, which makes human–robot interaction (HRI) a core concern for service design. Reviews of hospitality and tourism robotics research indicate rapid growth in publications and an emphasis on themes such as adoption antecedents, service quality perceptions, and the operational feasibility of robotics in hotels (Ivanov et al., 2019).

Service management research frames robots as frontline actors that reshape service encounters, customer roles, and employee tasks. The “frontline robot” perspective suggests that robot deployment can alter the boundaries of co-creation: customers may take on more self-service tasks, employees may shift toward supervision and exception handling, and the service organization may rely more on data-driven orchestration (Wirtz et al., 2018). These changes are especially consequential in hotels because service is delivered continuously and across multiple touchpoints, from booking and arrival to in-stay requests and post-stay communication.

Customer experience studies show that guests evaluate robotic encounters through multiple lenses, including perceived usefulness, enjoyment, security, social presence, and the fit between robot behavior and hospitality norms. For example, qualitative work on customer experiences with robotics highlights that novelty can create positive affect, but that breakdowns in interaction or perceived “coldness” can undermine satisfaction (Tung and Au, 2018). Trust is a central mechanism: travelers’ trust in intelligent service robots predicts their willingness to rely on robots, and trust is multifaceted, involving reliability, competence, benevolence, and integrity perceptions (Park, 2020; Tussyadiah et al., 2020).

Robot “smartness” introduces additional complexity. Highly autonomous or anthropomorphic robots can create better personalization and smoother interactions, but they may also trigger unease, heightened privacy concerns, or unrealistic expectations. Empirical research suggests that the degree of robot intelligence should be calibrated to the service context and customer expectations, implying non-linear effects (Schepers et al., 2022). As a result, managers must make strategic decisions about which service stages to automate and how to design handoffs between robots and humans.

From an organizational perspective, successful robotics adoption requires integration into routines and service scripts. Scale-development research on service robot integration willingness indicates that adoption depends on perceptions of benefits, compatibility, and organizational readiness (Lu et al., 2019). Moreover, conceptual frameworks emphasize a strategic choice between service enhancement and cost reduction objectives—each with different implications for brand identity, customer experience, and employee roles (Belanche et al., 2020; Belanche et al., 2021).

For this article, the critical insight is that robots and AI are not merely operational tools; they are intelligence-generating agents. Robots that navigate spaces, deliver items, and interact with guests produce behavioral traces (time stamps, routes, service requests, conversational logs) that can feed analytics and personalization. This shifts the theoretical framing from “robot adoption” to “capability building”: the integration of embodied automation with analytics can become a source of dynamic capability in sensing and shaping demand.

### 2.2 AI-enabled revenue management, pricing, and distribution

Revenue management has been a defining managerial innovation in hospitality. Classic yield-management principles emphasize that hotels can maximize revenue by allocating limited room inventory to customer segments with different willingness-to-pay profiles and booking patterns (Kimes, 1989). However, organizational and human factors remain critical: early discussions on yield management warn that success depends on aligning technology with service culture and frontline execution (Jones and Hamilton, 1992).

Revenue management subsequently expanded beyond room inventory to incorporate distribution strategy, marketing, and total revenue. Cross et al. (2009) describe a “renaissance” in which revenue management becomes integrated into hotel strategy and operations, emphasizing the role of analytics, segmentation, and price optimization across revenue streams. Contemporary scholarship suggests that the field is moving toward open pricing and one-to-one pricing, enabled by granular demand signals, customer data, and algorithmic decision support (Talón-Ballestero et al., 2022).

Digital distribution has altered the revenue management environment. Online travel agencies (OTAs), metasearch, and platform-based channels increase consumer price transparency and intensify rate competition, while also generating new data sources. Research on online booking behavior shows that the way prices are presented can influence willingness to book; customers respond differently to blended versus non-blended prices across multi-night stays (Noone and Mattila, 2009). Such findings underscore that revenue management is partly a behavioral science problem.

Dynamic pricing also raises fairness and trust issues. Using OTA data, Alderighi et al. (2022) show that dynamic pricing can reduce perceived fairness under certain conditions, implying that the economic logic of price discrimination must be balanced against psychological and reputational costs. These fairness dynamics become more salient as AI increases personalization and as customers increasingly compare prices across platforms and time. Moreover, the pursuit of extreme price personalization is constrained by customer acceptance and by increasing regulatory and competition-policy scrutiny of algorithmic pricing systems, including concerns about discrimination and tacit collusion (Calvano et al., 2020).

AI–robotics affects revenue management through multiple mechanisms. On the supply side, automation can change effective capacity by reducing service bottlenecks (e.g., faster room turnover due to robotic cleaning, more consistent delivery times for in-stay services) and by enabling more reliable service levels during labor shortages. On the demand side, robots can function as differentiating amenities, influencing willingness to pay, perceived innovativeness, and even perceived safety. Because trust and experience shape adoption (Park, 2020; Tung and Au, 2018), robotics can alter price elasticity in ways that traditional revenue management models do not capture.

Finally, AI-driven analytics can expand the set of controllable levers beyond price. Hotels can use predictive models to anticipate cancellations, identify high-value customers, optimize upgrade offers, and personalize packages. This reinforces the argument that the future of revenue management is “total value management,” where pricing, experience design, and operational orchestration are integrated (Cross et al., 2009; Talón-Ballestero et al., 2022).

### 2.3 Sustainability, smart operations, and circularity

Hospitality sustainability research has documented both progress and persistent challenges. Hotels have implemented initiatives such as towel and linen reuse, energy-saving lighting, water conservation, and sustainable sourcing. Yet, adoption varies by market, ownership type, and managerial commitment. Bohdanowicz (2006) found that hotels recognize environmental protection but face constraints shaped by economic and institutional context, implying that sustainability is embedded in broader systems.

Multinational chains have played a prominent role in scaling sustainability initiatives. Case-based research on Hilton’s European environmental program illustrates how environmental protection is operationalized through corporate policies, standards, and employee engagement (Bohdanowicz et al., 2011). From the consumer side, sustainable practices can influence guest satisfaction and intention to return, and effects may differ across nationalities and segments (Berezan et al., 2013). Systematic reviews call for research that links proactive environmental strategies to innovation capabilities and competitive advantage (Elkhwesky, 2022).

AI, robotics, and smart infrastructure enable a shift from static sustainability practices to dynamic optimization. Smart hospitality ecosystems can integrate building management systems, IoT sensors, and analytics to monitor resource consumption at granular levels (Buhalis and Leung, 2018). Predictive models can detect anomalies in energy usage, optimize HVAC settings based on occupancy patterns, and schedule maintenance to prevent equipment inefficiencies. Service robots can contribute by executing tasks with high precision (e.g., minimizing chemical and water use in cleaning) and by enabling demand-synchronized housekeeping. These mechanisms support the sustainability–profitability linkage by reducing resource intensity without necessarily reducing service quality.

However, sustainability increasingly depends on credible measurement and verification. Stakeholders—including corporate buyers, investors, regulators, and guests—scrutinize environmental claims, creating risks of perceived greenwashing. Blockchain technologies can complement AI by enabling immutable records of resource usage, supplier provenance, and ESG metrics. When combined with sensor data, blockchain can provide auditable sustainability evidence that strengthens trust and may translate sustainability efforts into market and financing benefits (Elkhwesky, 2022; Önder and Treiblmaier, 2018).

Circularity provides an additional lens. Hotels generate substantial waste streams (food waste, packaging, consumables) and rely on complex supply chains. AI can optimize procurement and inventory to reduce waste, while blockchain can enhance traceability and incentivize circular practices through tokenized credits or smart-contract-based supplier agreements. This integration is underexplored in hospitality research and offers fertile ground for theory and empirical work.

### 2.4 Hotel real estate and asset management: governance, value metrics, and technology

Hotels are distinctive real estate assets because cash flows depend heavily on operational decisions and brand positioning. Asset management therefore involves aligning operational strategies with owner and investor objectives and managing risks associated with demand volatility, capital expenditure, and contractual structures. Singh et al. (2012) describe the evolution of hotel asset management from troubleshooting toward continuous value maximization, reflecting the growing complexity of ownership and operating arrangements.

Performance measurement is central to asset management. While RevPAR remains widely used, it focuses on room revenue and ignores cost structure. Analyses comparing RevPAR and GOPPAR demonstrate that profit-based measures can diverge from revenue-based indicators and may better reflect the value implications of operational decisions (Lee et al., 2019). This matters for technology investments: AI and robotics may increase revenue, reduce costs, or both, but also may require additional CapEx and create new risks (e.g., cybersecurity).

Technology influences asset management at multiple levels. At the property level, AI-driven predictive maintenance can reduce downtime and extend asset life, while robotics can change labor models and service reliability. At the portfolio level, data aggregation can support benchmarking and risk analytics across assets. Smart hospitality research emphasizes interoperability as the foundation for such data integration (Buhalis and Leung, 2018).

Asset management also involves governance of owner–operator relationships. Management contracts often specify performance clauses, reporting requirements, and incentive structures. As hotels adopt AI-driven systems and robots, the “data layer” of operations becomes more consequential: who owns operational data, how performance is measured, and how disputes are resolved. Blockchain-based smart contracts could, in principle, automate parts of fee settlement or performance verification, potentially reducing agency costs and increasing transparency (Önder and Treiblmaier, 2018; Singh et al., 2012).

### 2.5 Blockchain technologies in hospitality and real estate: trust, smart contracts, and tokenization

Blockchain has been discussed in tourism as a technology that can reshape transactions and governance. Önder and Treiblmaier (2018) propose that blockchain can enable disintermediation, improve transparency, and facilitate new governance mechanisms through immutable records and programmable rules. Kwok and Koh (2019) argue that blockchain may be a watershed for tourism development, particularly through applications in payments, loyalty, identity, and peer-to-peer marketplaces.

In hotel ecosystems, blockchain’s value proposition is best understood as a coordination and verification layer rather than as a stand-alone system. Potential hospitality-relevant functions include:

- Identity, credentialing, and consent management across touchpoints (booking, check-in, loyalty enrollment, personalization).
- Transaction and commission audit trails across distribution partners (OTAs, wholesalers, metasearch) and automated settlement through smart contracts.
- Interoperable loyalty ecosystems (tokenized points, cross-brand redemption) with transparent issuance and redemption rules.
- Sustainability traceability (resource consumption logs, supplier provenance, verified ESG metrics) to reduce greenwashing risk.
- Owner–operator contracting support, including automated performance clause verification and reporting governance.
- Tokenization of value (e.g., loyalty value, carbon credits, fractional investment interests) that can create new incentive mechanisms and financing options.

These functions become more valuable when combined with AI and robotics. AI generates predictions and autonomous actions; blockchain provides an auditable record of transactions and decisions. Together, they can support accountable automation, where algorithmic decisions are explainable, traceable, and contractually embedded. However, blockchain implementations vary widely, and trade-offs exist between scalability, governance, privacy, and energy use. These trade-offs are especially salient in a sustainability-focused hospitality context.

### 2.6 Synthesis and research gaps

The reviewed literature indicates strong progress within each stream but limited integration across them. Robotics research has deepened understanding of trust and experience mechanisms, but rarely connects those mechanisms to pricing and asset value outcomes. Revenue management research has advanced dynamic and open pricing, but often treats service automation as exogenous to demand and capacity. Sustainability research shows the strategic importance of environmental initiatives, yet seldom theorizes how digital infrastructures enable measurement, optimization, and verification at scale. Asset management research highlights governance and value maximization but has not fully incorporated AI-enabled operational intelligence and blockchain-based contracting.

Consequently, hospitality scholars lack a holistic theory of how AI–robotics and blockchain jointly reshape hotel decision making and performance. The framework suggests that capability building constitutes the primary integration point. In particular, digital operational and market-intelligence capabilities provide the means to synchronize revenue management, sustainability, and asset management decision-making, replacing fragmented, domain-specific optimization. The next sections develop a theory-driven conceptual model and propositions to address this gap. Put differently, the core gap is not technological (whether hotels can adopt AI, robots, or blockchain), but theoretical-organizational and ecosystemic: the field lacks models that explain how digitally enabled coordination, governance, and capability orchestration translate into sustainable value under interdependence and complex governance.

## 3 Methodology

### 3.1 Integrative literature synthesis and theory-building logic

This paper is a conceptual and theory-building contribution rather than an empirical test. Conceptual articles are particularly useful when a phenomenon is emergent and multi-disciplinary, and when existing evidence is distributed across research communities. The convergence of AI–robotics, blockchain, revenue management, sustainability, and hotel real estate exhibits these characteristics (Ivanov et al., 2019; Önder and Treiblmaier, 2018).

An integrative literature synthesis approach was employed that combines problem-driven scoping, thematic analysis, and relational theorizing. First, the phenomenon around four focal areas is scoped: (a) AI and service robotics in hospitality service systems; (b) revenue management, dynamic pricing, and distribution; (c) sustainability and operational efficiency; and (d) hotel real estate and asset management. Blockchain scholarship from tourism and service ecosystems is also included as a complementary lens (Kwok and Koh, 2019).

Second, a structured thematic aggregation of mechanisms emphasized in each stream was conducted. For example, robotics research highlights adoption, trust, and HRI; revenue management emphasizes forecasting, segmentation, and price fairness; sustainability research emphasizes environmental program design, guest responses, and strategic integration; and asset management emphasizes governance, value metrics, and agency relationships (Alderighi et al., 2022; Singh et al., 2012).

Third, a concept matrix is developed that links mechanisms to outcomes and to the three hotel decision domains central to this paper: revenue management, sustainability and operations, and real estate/asset management. This mapping process revealed a unifying mediator: digital operational and market-intelligence capabilities. These capabilities capture the ability to sense (collect and interpret signals), predict (forecast demand and operational states), orchestrate (coordinate resources and service scripts), and verify/coordinate across stakeholders through trustworthy records and programmable rules. Smart hospitality scholarship provides the ecosystem foundation for these capabilities (Buhalis and Leung, 2018; Buhalis et al., 2023).

Finally, this capability is used to develop a conceptual model and propositions that can guide future empirical research. The propositions are directional statements about relationships between constructs and are intentionally formulated to be testable using a range of methods: longitudinal econometrics, experiments, simulation, case studies, and design science. Moderators are also explicitly identified that reflect responsible innovation concerns, including governance, ethics, privacy, and cybersecurity, as well as organizational readiness and institutional context.

### 3.2 Scope, unit of analysis, and global applicability

The model is designed for the global hotel sector and is applicable to varied property types (limited-service through luxury), ownership structures (owned/operated, managed, franchised, leased), and portfolio strategies. Hotels are conceptualized as embedded in a multi-actor ecosystem: guests, employees, operators, owners/investors, brands/franchisors, technology vendors, online intermediaries, and regulators (Buhalis et al., 2023).

While propositions are stated at the firm or property level, it is recognized that some mechanisms operate at multiple levels. For example, trust in robots is an individual-level mechanism that aggregates to demand outcomes; interoperability is a system-level condition; and asset value is typically assessed at the property or portfolio level. Future empirical work should therefore consider multi-level research designs and cross-cultural comparisons, given evidence that trust and acceptance vary across contexts (Park, 2020).

## 4 Conceptual model and propositions

### 4.1 Theoretical foundations

Three theoretical lenses guide the conceptual model. First, service ecosystem thinking emphasizes that value creation in hospitality emerges from interactions among actors, technologies, and institutional arrangements. In smart hospitality ecosystems, interoperability and networked collaboration enable co-created value across stakeholders (Buhalis et al., 2023; Wirtz et al., 2018).

Second, a dynamic capability perspective clarifies how hotels convert technology resources into performance. AI–robotics and blockchain are treated as enabling resources that, when integrated, create higher-order capabilities in sensing, prediction, orchestration, and trusted coordination. These capabilities allow hotels to adapt revenue management, sustainability operations, and asset decisions under uncertainty and change. In this framing, AI–robotics and blockchain are enabling resources; the explanatory core of the framework lies in the mediating digital operational and market-intelligence capability that orchestrates these resources into sustainable outcomes. This emphasis shifts attention away from technology adoption alone and reduces the risk of technodeterministic interpretation by highlighting organizational processes, governance, and ecosystem coordination.

Third, transaction cost and agency perspectives from hotel asset management highlight governance challenges created by the separation of ownership and operations. Asset managers seek to reduce agency costs and align incentives through reporting, benchmarking, and contract design (Singh et al., 2012). Blockchain-based smart contracts and auditable records can be theorized as governance technologies that may complement or substitute traditional monitoring mechanisms.

### 4.2 Model description

**FIGURE 1:**
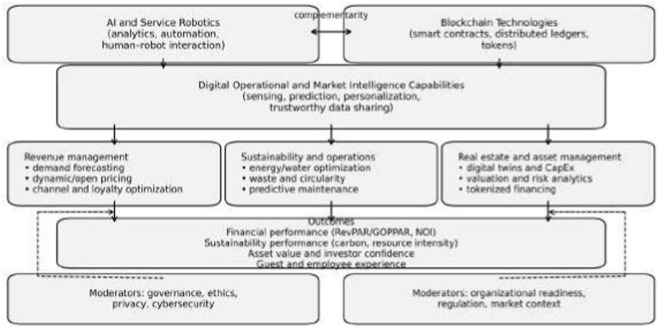
Conceptual model of blockchain-enabled AI–robotics capabilities for sustainable hotel revenue management, operational sustainability, and asset value.

Figure 1 depicts the conceptual model. AI–robotics capability captures a hotel’s ability to deploy AI models and robotic/automated systems to sense environments, predict demand and operational needs, and execute tasks while maintaining service quality. Blockchain capability captures the ability to use distributed ledgers, smart contracts, and tokenization to create immutable records, automate contractual clauses, manage digital identity and consent, and enable transparent value transfer (Önder and Treiblmaier, 2018). In practice, this encompasses AI-driven revenue management and demand-forecasting systems, conversational agents for service support and recovery, and physically embodied robots for delivery, cleaning, and concierge functions (Belanche et al., 2020; Ivanov et al., 2019). In hotel ecosystems, blockchain capability may be expressed through smart-contract-based commission settlement across distribution partners, interoperable tokenized loyalty schemes, and verifiable ESG data logs shared across owners, operators, and investors (Kwok and Koh, 2019; Önder and Treiblmaier, 2018).

The central mediator is digital operational and market-intelligence capability, defined as the hotel’s ability to integrate data from service encounters, operations, and ecosystem partners to support sensing, prediction, personalization, orchestration, and trustworthy coordination (Buhalis and Leung, 2018; Mariani and Baggio, 2022). This capability influences three interdependent decision domains: revenue management, sustainability and operations, and real estate/asset management. These domains jointly determine outcomes in financial performance, sustainability performance, asset value, and stakeholder experience.

Two sets of moderators shape the strength and direction of relationships. Governance, ethics, privacy, and cybersecurity moderate whether automation and data sharing create trust or resistance. Organizational readiness, regulatory regimes, and market context moderate feasibility and adoption speed, highlighting the global nature of technology diffusion in hospitality. For example, transparent disclosure and human-in-the-loop oversight can strengthen trust in automated service, whereas opaque personalization, weak consent management, or poor cybersecurity can amplify perceived risk and resistance (Schepers et al., 2022; Tussyadiah et al., 2020).

### 4.3 Research propositions

Building on the literature synthesis and theoretical lenses, ten propositions are proposed, organized by the three focal decision pathways. In each case, propositions emphasize mechanisms and boundary conditions rather than effect sizes.

#### 4.3.1 Revenue management pathway

Proposition 1 (P1). AI–robotics capability increases revenue management effectiveness by improving demand sensing, forecasting, and operational reliability, enabling more responsive dynamic/open pricing decisions. AI systems can process high-frequency demand signals (search data, review sentiment, booking patterns), while robots can stabilize operational performance (e.g., service delivery times, room readiness). Together, these improvements increase the feasibility of data-driven pricing approaches that depend on accurate forecasts and reliable service capacity (Cross et al., 2009; Kimes, 1989; Talón-Ballestero et al., 2022).

Proposition 2 (P2). The relationship between AI–robotics capability and revenue outcomes is mediated by guest experience and trust in automated service, such that positive robot experiences increase willingness to pay and reduce demand volatility. Service robots can differentiate the offering, but only when customers perceive them as useful and trustworthy. Empirical work highlights the role of trust, competence, and perceived control in shaping acceptance and evaluations of robot-delivered service (Park, 2020; Tung and Au, 2018; Tussyadiah et al., 2020). These psychological mechanisms influence willingness to pay and price elasticity, connecting HRI research to revenue outcomes.

Proposition 3 (P3). Blockchain capability improves net revenue capture by reducing transaction frictions in distribution and loyalty (e.g., commission reconciliation, attribution, and redemption) through auditable records and smart-contract-based settlement. Hotels operate within multi-party distribution networks that create reconciliation costs and disputes. Distributed ledgers can provide shared records of bookings, cancellations, and commissions, while smart contracts can automate settlement under agreed rules. Tourism research positions blockchain as enabling transparency and reducing intermediation, suggesting that these mechanisms can translate into improved net revenue outcomes when distribution costs decline (Kwok and Koh, 2019; Önder and Treiblmaier, 2018).

Proposition 4 (P4). Perceived price unfairness negatively moderates the effect of AI-enabled dynamic/open pricing on revenue performance; blockchain-enabled transparency and verifiable pricing rules can weaken (but not eliminate) this negative moderation. Behavioral pricing research shows that dynamic pricing can reduce perceived fairness under conditions of salient price dispersion (Alderighi et al., 2022). Online contexts further shape perceived fairness via price presentation strategies (Noone and Mattila, 2009). Blockchain can support transparent, auditable pricing rules and loyalty value attribution, which may make pricing logic more explainable and credible, thereby partially mitigating fairness concerns. However, transparency can also increase price comparisons, suggesting that the net effect depends on design choices and communication.

#### 4.3.2 Sustainability and operations pathway

Proposition 5 (P5). AI–robotics capability improves sustainability performance by enabling real-time resource optimization and predictive maintenance, reducing energy, water, and material intensity per occupied room. Smart hospitality ecosystems integrate sensors and analytics to optimize building operations and service processes (Buhalis and Leung, 2018). Predictive maintenance reduces resource waste associated with inefficient equipment, while robotics can execute tasks with precision and consistency. These mechanisms align with calls to shift sustainability research from isolated practices to dynamic operational strategies (Elkhwesky, 2022).

Proposition 6 (P6). Interoperability in smart hospitality ecosystems positively moderates the sustainability impact of AI–robotics capability, because integrated data flows enable system-level optimization across departments and properties.

Interconnectivity and interoperability are foundational to smart hospitality, enabling data exchange between internal systems and external partners (Buhalis and Leung, 2018; Buhalis et al., 2023). When interoperability is high, resource optimization can occur across housekeeping, laundry, maintenance, and food-and-beverage, generating larger sustainability gains than isolated technology deployments.

Proposition 7 (P7). Blockchain capability strengthens the relationship between sustainability practices and stakeholder outcomes (guest trust, investor confidence, and corporate contracting) by enabling auditable sustainability records and supply-chain traceability.

Sustainable practices can influence guest satisfaction and intentions (Berezan et al., 2013), but stakeholder responses depend on credibility. Systematic reviews emphasize the strategic importance of proactive environmental strategies and the need to reduce greenwashing risk (Elkhwesky, 2022). Blockchain can provide traceable records of procurement, waste handling, and resource consumption, potentially increasing the reputational and financial benefits of sustainability efforts.

#### 4.3.3 Real estate and hotel asset management pathway

Proposition 8 (P8). AI–robotics capability improves hotel asset management effectiveness by enabling predictive CapEx planning and operational risk analytics, increasing NOI stability and supporting long-term asset value.Asset managers seek to maximize hotel values through continuous oversight and strategic interventions (Singh et al., 2012). AI-driven predictive analytics can anticipate refurbishment needs and quantify the revenue and cost impacts of CapEx timing, while robotics can change labor models and service reliability. Because profit-based performance measures can diverge from revenue indicators, these analytics should be evaluated in relation to both RevPAR and GOPPAR outcomes (Lee et al., 2019).

Proposition 9 (P9). Blockchain capability reduces agency costs in owner–operator relationships by enabling smart-contract enforcement of performance clauses and transparent data-sharing arrangements.

The separation of ownership and operations creates information asymmetries and incentive conflicts that hotel asset management seeks to address (Singh et al., 2012). Blockchain-based ledgers can create shared performance records, and smart contracts can automate fee settlement or trigger contractual actions based on verified metrics. These governance mechanisms align with blockchain’s theorized role in enhancing transparency and programmable governance (Önder and Treiblmaier, 2018).

Proposition 10 (P10). The combined deployment of AI–robotics and blockchain creates complementary value for hotel real estate by enabling new financing and investment structures (e.g., tokenized interests linked to verified operational and ESG metrics), contingent on regulatory and governance fit.

Tokenization can, in principle, broaden investor participation and enable new liquidity mechanisms, while AI-driven operational intelligence provides the information needed to price and manage such instruments. However, real estate tokenization raises legal, governance, and fiduciary considerations, and its feasibility depends on local regulation and institutional acceptance. The proposition therefore emphasizes complementarity and boundary conditions rather than deterministic outcomes (Kwok and Koh, 2019).

## 5 Findings

Although this article is conceptual, the model development yields several “findings” in the form of integrative theoretical insights and propositions that clarify how technologies can influence hotel value creation. These conceptual findings are summarized as four interrelated insights.

Finding 1: Technology complementarity is central. AI–robotics and blockchain generate the greatest strategic value when treated as complementary infrastructures. AI increases the autonomy and scale of decisions (forecasting, personalization, automation), while blockchain increases auditability, trust, and enforceability of ecosystem interactions. This complementarity underpins Propositions 1–4 (revenue) and Propositions 7–10 (verification and governance).

Finding 2: Revenue management becomes an orchestration problem rather than a pure pricing problem. Traditional yield management focused on allocating inventory under fixed operational constraints (Kimes, 1989). Under AI–robotics, operational constraints themselves become partially controllable: automation can change service capacity, reduce variability, and enable new service bundles that alter willingness to pay. Consequently, revenue management should be conceptualized as integrated orchestration of price, experience, and operational reliability (Cross et al., 2009; Talón-Ballestero et al., 2022).

Finding 3: Sustainability value depends on optimization and verification. Sustainability research shows that environmental initiatives can affect guest outcomes and are increasingly part of hotel strategy (Berezan et al., 2013; Elkhwesky, 2022). Our model clarifies that AI and robotics primarily contribute through dynamic optimization (resource efficiency, predictive maintenance), while blockchain primarily contributes through credibility and traceability (auditable ESG data, provenance records). These mechanisms jointly support stakeholder trust and can translate sustainability performance into financial and reputational returns. To clarify outcome pathways, efficiency based environmental benefits (e.g., energy, water, and waste reductions) were distinguished from social sustainability outcomes (e.g., job quality, inclusion, and guest well-being) and from long-term value creation. Environmental efficiency gains can be immediate, but social legitimacy and long-term asset value depend on governance choices, stakeholder communication, and how technology is embedded in service design.

Finding 4: Asset management integrates and “capitalizes” operational technology effects. Hotel asset management scholarship emphasizes the role of governance and continuous value maximization (Singh et al., 2012). The model shows that technology adoption decisions in revenue management and sustainability are also asset-management decisions because they change NOI stability, CapEx trajectories, and risk profiles. The distinction between RevPAR and GOPPAR reinforces that profitability and cost structures are critical pathways through which technology influences value (Lee et al., 2019).

Collectively, these findings support a shift in both research and practice: from assessing isolated technologies to assessing integrated capability architectures and governance mechanisms. The next section discusses implications and boundary conditions in more depth.

## 6 Discussion

### 6.1 Integrating revenue, sustainability, and asset value

The proposed model suggests that hotels should treat revenue management, sustainability, and asset management as mutually dependent. For example, robotics-driven process improvements can reduce labor hours and improve service reliability, affecting both GOPPAR and guest experience. Sustainability-oriented resource optimization can reduce operating costs and support corporate contracting, which in turn stabilizes NOI and asset value. These cross-domain effects imply that siloed governance structures (separate RM, sustainability, and ownership functions) may limit the benefits of technology adoption.

### 6.2 Blockchain as institutional infrastructure for accountable automation

In many hotel ecosystems, stakeholders distrust data and dispute attribution (e.g., who influenced a booking, whether sustainability claims are valid, whether performance metrics are correctly calculated). Blockchain can support a shared record of transactions and metrics, potentially reducing disputes and enabling more automated contracting. This is particularly relevant when AI systems make pricing or operational decisions that affect multiple parties. However, blockchain introduces its own governance challenges: who controls the protocol, how privacy is protected, and how off-chain data integrity is ensured. Thus, blockchain value depends on institutional alignment, not merely technical deployment.

### 6.3 Ethical, privacy, and fairness issues

As AI and robots become more pervasive, ethical considerations move from abstract principles to operational decisions. First, privacy and consent are central because robots and IoT systems collect data in physical spaces, including potentially sensitive information. Second, algorithmic pricing raises fairness concerns, as consumers may perceive personalized prices as discriminatory or manipulative (Alderighi et al., 2022). Third, transparency is a double-edged sword: while explainability can increase trust, greater transparency can also intensify price comparison and encourage strategic consumer behavior. Blockchain-based transparency therefore requires careful design to balance accountability with competitive and privacy needs.

### 6.4 Workforce and social implications

Automation changes job designs and skill requirements. Service robots may reduce certain repetitive tasks but increase demand for roles related to supervision, maintenance, data interpretation, and service recovery. The risk is not only job displacement but also potential deskilling or increased work stress if employees must manage complex technology without adequate training. Service management research emphasizes that robots reconfigure the frontline and the customer role, suggesting that human–robot collaboration design should be a strategic priority (Wirtz et al., 2018). Intensive technological integration can also produce unintended effects. Excessive automation may erode the human touch that differentiates hospitality if robots substitute for relational service rather than augment it. Likewise, over-reliance on data-driven systems can create operational fragility when data are biased, systems fail, or cyber incidents disrupt service. These risks reinforce the need for human-centered design, redundancy, and clear escalation pathways for service recovery.

### 6.5 Global and institutional heterogeneity

Hospitality is globally diverse in labor costs, cultural norms, infrastructure quality, and regulation. Trust in robots and willingness to accept automated service vary across cultures (Park, 2020), while privacy regimes and AI regulation differ across jurisdictions. Similarly, blockchain regulation and the legality of tokenized assets vary widely. Therefore, the diffusion and outcomes of these technologies will be uneven, reinforcing the need for cross-cultural and comparative research designs.

### 6.6 Future trends

Several developments are likely to intensify the relevance of this framework. First, open pricing and personalization will expand as hotels integrate more data sources, but fairness and privacy constraints will remain central. Second, sustainability reporting requirements and investor pressure for ESG transparency will increase the value of verifiable data infrastructures, potentially accelerating blockchain-based ESG applications. Third, real estate finance innovation, including tokenization experiments, may increase, particularly if linked to verified performance data. Finally, advances in generative AI and conversational agents may blur boundaries between robots, virtual assistants, and revenue-management interfaces, making governance and accountability even more critical. These trajectories will be shaped by increasing regulatory scrutiny of algorithmic personalization and pricing, as well as by evolving norms around transparency and responsible AI.

## 7 Implications

### 7.1 Implications for the hospitality industry

Revenue management leaders should collaborate with operations and technology teams to incorporate automation-driven capacity changes into forecasting and pricing. For example, if robotic cleaning reduces room turnover time variance, RMS forecasts and overbooking limits may be recalibrated. Similarly, if robots improve in-stay service responsiveness, hotels can test pricing of “service reliability” attributes through bundles or tiered offerings (Cross et al., 2009; Talón-Ballestero et al., 2022).

Hotels should proactively manage fairness and transparency in dynamic pricing. Behavioral research suggests that dynamic pricing can harm fairness perceptions under certain conditions (Alderighi et al., 2022). Managers should evaluate not only algorithmic performance but also communication, explainability, and consistency with brand promises. Blockchain-enabled audit trails can support accountability, but transparency should be designed to avoid unintended consequences such as increased price competition.

Sustainability managers should treat AI and IoT as core tools for continuous resource optimization and should integrate sustainability KPIs into operational dashboards. For external reporting and stakeholder trust, blockchain-based ESG data infrastructures can support traceability and credibility, particularly for corporate customers and investors demanding verified sustainability metrics (Elkhwesky, 2022).

Owners and asset managers should evaluate technology through an asset-value lens. Investments in AI, robotics, and blockchain should be assessed for impacts on NOI stability, risk, CapEx needs, and exit value, not just short-term RevPAR. Smart-contract experiments may be most feasible initially for internal governance (automated reporting, performance clause verification) before extending to broader financing or tokenization use cases (Singh et al., 2012).

Human capital strategies are essential. Hotels should invest in reskilling and redesign of roles to support human–robot collaboration, service recovery, and technology oversight. Ethics, privacy, and cybersecurity governance should be built into technology programs from the start, given the sensitivity of guest data and the reputational risks of breaches or biased algorithms (Schepers et al., 2022).

### 7.2 Implications for hospitality, tourism, and real-estate academia

The model invites integrative theorizing across domains that are often studied separately. Scholars can connect micro-level HRI mechanisms (trust, perceived control, social presence) to macro-level outcomes (pricing power, NOI stability, asset value). This integration can enrich both service robotics and revenue management theory (Park, 2020; Talón-Ballestero et al., 2022).

Researchers should pursue multi-level and longitudinal designs. Technology effects may unfold over time as guests and employees learn, as algorithms improve, and as organizational routines adapt. Comparative designs across countries and ownership structures can reveal how regulation, culture, and governance shape adoption and outcomes.

Methodologically, hospitality research can leverage new data sources created by these technologies, including robot logs, IoT sensor data, and potentially blockchain transaction records. This opens opportunities for causal inference, process mining, and simulation, but also raises ethical and privacy challenges that require robust research governance.

Finally, the framework highlights a need for validated measurement of emerging constructs (e.g., blockchain capability, auditability, smart-contract governance) in hospitality contexts, analogous to prior scale development for robot integration willingness (Lu et al., 2019).

## 8 Suggestions for future research

The propositions developed here provide a foundation for a broad research agenda. Priority directions are outlined, organized around the focal domains and cross-cutting governance issues.

1. Pricing and experience co-design. Future research should test how robot-enabled service attributes affect willingness to pay, price elasticity, and booking behavior. Experiments could manipulate the presence, role, and transparency of robots in service delivery and examine downstream impacts on purchase intentions under different pricing schemes. Such work would integrate HRI insights (trust and perceived control) with revenue management outcomes (Park, 2020; Tung and Au, 2018).
2. Algorithmic fairness and explainability in hospitality pricing. Researchers can investigate which forms of algorithmic transparency increase trust without triggering adverse competitive dynamics. Studies could compare traditional explanations (e.g., demand-based pricing) with blockchain-enabled verifiable rules and assess effects on perceived fairness, satisfaction, and loyalty (Alderighi et al., 2022; Noone and Mattila, 2009).
3. Blockchain-enabled distribution governance. Empirical work should examine whether distributed ledgers reduce reconciliation costs and disputes in multi-party distribution. Research questions include: Which partners are willing to participate? How does governance of the ledger affect power dynamics? What data should be shared on-chain versus off-chain? Case studies and action research with industry consortia could be especially valuable.
4. Verified sustainability and market outcomes. Researchers should test whether verifiable sustainability metrics influence corporate contracting, guest willingness to pay, and investor financing terms. For example, do blockchain-verified ESG metrics reduce perceived greenwashing and increase trust compared with self-reported claims? Quasi-experimental designs could exploit regulatory changes in sustainability reporting to evaluate causal effects (Elkhwesky, 2022).
5. Digital twins, CapEx timing, and resilience. Asset management research can examine how AI-driven digital twins support refurbishment decisions, predictive maintenance, and resilience planning under climate risks and demand shocks. Linking digital twin adoption to NOI stability and valuation measures would strengthen the bridge between operations and real estate finance (Lee et al., 2019).
6. Smart contracts in owner–operator relationships. Scholars can explore how smart contracts alter monitoring, incentives, and dispute resolution between owners, operators, and brands. Comparative studies across contract types (management, franchise, lease) can identify where automation is feasible and where human judgment and negotiation remain essential (Singh et al., 2012).
7. Workforce transition and service recovery. Research should study how employee roles evolve in robot-enabled hotels, including training needs, job satisfaction, and service recovery processes. Longitudinal qualitative studies could examine how service culture changes as robots become routine, while quantitative studies could test effects on employee retention and guest complaints.
8. Cybersecurity and risk management. As hotels become more connected and automated, cybersecurity risks increase. Future research should develop hospitality-specific frameworks for cyber risk in smart hotels, including how blockchain architectures influence attack surfaces. This topic is closely linked to asset value because cyber incidents can create operational disruption and reputational damage.
9. Cross-cultural and institutional comparisons. Because hospitality is global, researchers should compare adoption and outcomes across countries with different labor markets, cultural norms, and regulatory regimes. Such work can refine boundary conditions in the conceptual model, especially regarding trust, privacy, and acceptance of tokenized assets.

## 9 Conclusion

This conceptual article integrates robotics/AI and blockchain technologies in hospitality with a specific focus on revenue management, sustainability, and hotel real estate/asset management. By theorizing how AI–robotics and blockchain jointly build digital operational and market-intelligence capabilities, the proposed model explains pathways to improved financial performance, sustainability outcomes, and long-term asset value, while highlighting governance and contextual boundary conditions. The propositions and research agenda offered here aim to support scholars and practitioners in designing, evaluating, and governing technology-enabled hotel ecosystems in a rapidly evolving global environment.

## Data Availability

The original contributions presented in the study are included in the article/supplementary material, further inquiries can be directed to the corresponding author.
